# Cytoprotective Activity of Natural and Synthetic Antioxidants

**DOI:** 10.3390/antiox9080713

**Published:** 2020-08-06

**Authors:** Kateřina Valentová

**Affiliations:** Laboratory of Biotransformation, Institute of Microbiology of the Czech Academy of Sciences, Vídeňská 1083, 14220 Prague, Czech Republic; kata.valentova@email.cz

Numerous chronic diseases including cancer, cardiovascular, chronic respiratory or neurodegenerative diseases, diabetes mellitus, retinal damage, and others are associated with oxidative stress. Therefore, various natural and synthetic antioxidants are proposed for the prevention and treatment of such diseases [1]. Cellular protection against oxidative and electrophile toxicities (chemoprevention) can be provided either by redox-active, short-living direct antioxidants or indirect antioxidants having both anti- and prooxidant activity, activating the Keap1/Nrf2/ARE pathway, which results in the transcriptional induction of cytoprotective proteins [2,3,4]. Individual antioxidant protective systems in humans cooperate in a complex functional interplay; cytoprotective proteins are involved in the synthesis and/or regeneration of direct antioxidants, which in turn are often required for the catalytic functions of cytoprotective proteins, which are the real “antioxidant” effectors [3,5]. Direct antioxidant therapy often failed in clinical trials and recent studies mostly confirm the involvement of several cell signaling pathways in cytoprotection from oxidative stress [6,7,8].

As further investigation into this subject is likely to provide renewed insight into the molecular mechanisms of oxidative stress in chronic diseases, the aim of this Special Issue was to compile up to date original research dealing with the cytoprotective activity of natural antioxidants and inducers of cytoprotective proteins from plants or microorganisms, but also (semi)synthetic molecules and especially pleiotropic agents with protective effects in the abovementioned chronic diseases. Studies focusing on the molecular mechanism of action of cytoprotective agents were particularly welcome.

In line with this, one of the two reviews in this Issue highlights the critical role of the Keap1/Nrf2 pathway, its modulation by natural compounds, and the growing interest in clinical applications of the Nfr2 activators in, e.g., endocrinology/metabolism, cardiology, and nephrology [9]. As a response to the urgent need for the treatment of severe acute respiratory syndrome coronavirus 2 (SARS-CoV-2) disease (COVID-19), the second review deals with the potential cytoprotective activity of ozone therapy in this viral infection via decreasing organ damage mediated by inflammation and oxidative stress [10].

To more easily monitor the cytoprotective effect of antioxidants, a new quantitative assay called Anti Oxidant Power 1 (AOP1) was developed, which specifically measures reactive oxygen species (ROS) and/or free radical scavenging effects inside living cells using Light Up Cell System (LUCS) technology allowing fine monitoring of ROS production inside live cells. The assay was used to compare intracellular antioxidant/prooxidant efficacy of 15 well-known antioxidants with different hydrophilic/lipophilic properties [11].

An important line of research deals with possible protection against toxicity induced by various drugs using both natural and synthetic agents. Treatment of oncological conditions with chemotherapeutics such as the platinum-based antineoplastic medications often results in toxic side-effects towards non-cancer tissues and cells [12]. Berberin containing the methanolic root extract of *Berberis vulgaris* L. displayed both a prophylactic and curative protective effect against nephrotoxic, hepatotoxic, and hyperlipidemia effects of cisplatin in vivo in male albino Wistar rats [13]. On the other hand, calmangafodipir (proprietary name: PledOx^®^), an analogue of manganese superoxide dismutase mimetic, chelating agent, and intravenous magnetic resonance imaging agent mangafodipir showed preventive and protective effects against oxaliplatin-induced neurotoxicity in BALB/c mice [14].

A growing number of studies deal with the mechanism of action of cytoprotection. The cytoprotective mechanism of natural anthocyanin delphinidin against oxidative stress induced by H_2_O_2_ was investigated in human chondrocytes where it inhibited reactive oxygen species (ROS)-induced apoptosis via activation of Nrf2 and nuclear factor κB (NF-κB) and activated cytoprotective autophagy showing potential in the treatment of osteoarthritis [15]. Embelin, a plant natural product from *Lysimachia punctata* and *Embelia ribes* fruit with quinone and hydroquinone functional groups plus a long hydrophobic tail completely abolished the superoxide radical generated in situ with hydrodynamic voltammetry. Moreover, it exhibited cytoprotective activity in THP-1 human leukemic monocytes and BV-2 mice microglia probably thanks to its long alkyl tail enabling its insertion in cell membranes [16]. The mechanism of anti-inflammatory and antioxidant effects of aspirin on hyperoxia-induced acute lung injury was studied in NF-κB–luciferase transgenic mice. Pretreatment with aspirin significantly reduced the protein levels of phosphorylated protein kinase B, NF-κB, and tumor necrosis factor α, indicating that aspirin reduces NF-κB activation [17].

A few publications were devoted to the effect of natural compounds in the form of complex but well-characterized extracts. Thus, the essential oil from feijoa (*Acca sellowiana*) fruit peal containing mostly sesquiterpenes showed strong antioxidant and free radical scavenging activity, cytoprotective activity on lymphocytes pre-treated with 100 μM *tert*-butylhydroperoxide (*t*-BOOH), as well as a decrease in intracellular ROS, induced by *t*-BOOH on erythrocytes and antimicrobial and antifungal activities against *Staphylococcus aureus* and *Candida albicans*, respectively [18]. *Vitis vinifera* var. Fetească neagră tendrils and leaves extracts, intended to be used in oral hygiene products recommended in periodontal disease, displayed a cytoprotective effect against nicotine-induced cytotoxicity and anti-inflammatory activity in human gingival fibroblasts [19]. *Viburnum opulus* berry phenolic extracts were able to decrease the uptake of glucose, free fatty acids, and accumulation of lipid droplets in Caco-2 cells without affecting their viability, followed by a decrease in the CD36/FAT gene expression, without influence on the GLUT2 and PPARα levels. Furthermore, the extracts revealed strong chemo-preventive activity against oxidative stress induced chemically by *t*-BOOH, as well as against DNA damage through the induction of DNA repair after cell exposition to methylnitronitrosoguanidine and H_2_O_2_ [20]. In a subsequent study, *V. opulus* fruit fresh juice and a phenolic-rich fraction were able to decrease intracellular oxidative stress in mice insulinoma MIN6 cells, induced glucagon-like peptide-1 secretion in the presence of an elevated glucose concentration, and inhibited in vitro activity of the dipeptidyl peptidase-4 [21].

Several publications in this Special Issue study flavonolignans from silymarin, an extract from the fruits of *Silybum marianum*. Twenty-six commercially available silymarin preparations, natural silymarin from Sigma Aldrich, and a model mixture of pure flavonoid/flavonolignans mimicking the silymarin composition, all analyzed by U-HPLC-HRMS/MS, were compared using biochemical (2,2′-azino-*bis*-(3-ethylbenzothiazoline-6 sulfonic acid) (ABTS), oxygen radical absorption capacity (ORAC), and 2,2-diphenyl-1-picrylhydrazyl (DPPH)) and cellular (CAA) antioxidant tests and significant differences in the antioxidant capacity of the supplements were observed [22]. Antioxidant activities of pure stereomers A and B of silybin and 2,3-dehydrosilybin, their racemic mixtures, pure silychristin A, and its derivatives (anhydrosilychristin, isosilychristin, and 2,3-dehydrosilychristin A) were investigated by using ORAC and CAA assays. Moreover, their anti-inflammatory activity was studied in macrophages and multidrug resistance modulation as inhibition of P-glycoprotein and sensitization of doxorubicin-resistant ovarian carcinoma cells overproducing P-glycoprotein was detected and the effect on related gene expression revealed a distinct mechanism of action for the individual compounds [23,24]. Last but not least, new potential targets of silymarin constituents silybin and dehydrosilybin as dual inhibitors of BRAF and the Hedgehog pathway receptor Smoothened (SMO), two major targets in anticancer therapy, were identified in silico and confirmed in vitro [25].

The content of the Special Issue clearly shows that cytoprotection by anti- and prooxidant active molecules is still a hot topic. However, for effects demonstrated in cell cultures other than those derived from the gastrointestinal tract, the bioavailability and metabolism of the active compounds often could not be taken into consideration. Therefore, future studies should be directed towards the effects of the metabolites likely to be present in blood plasma.
